# Effects of a switch from tenofovir- to abacavir-based antiretroviral therapy, with or without atazanavir, on renal function

**DOI:** 10.7448/IAS.19.1.20995

**Published:** 2016-09-09

**Authors:** Silvia A Guillemi, Sean H Ling, Julia S Dahlby, Benita Yip, Wendy Zhang, Mark W Hull, Viviane Dias Lima, Robert S Hogg, Ronald Werb, Julio S Montaner, Marianne Harris

**Affiliations:** 1 British Columbia Centre for Excellence in HIV/AIDS, St. Paul's Hospital, Vancouver, BC, Canada; 2 Department of Family Practice, Faculty of Medicine, University of British Columbia, Vancouver, BC, Canada; 3 Division of AIDS, Department of Medicine, Faculty of Medicine, University of British Columbia, Vancouver, BC, Canada; 4 Faculty of Health Sciences, Simon Fraser University, Burnaby, BC, Canada

**Keywords:** HIV, tenofovir, abacavir, atazanavir, renal, renal dysfunction, HAART

## Abstract

**Introduction:**

Tenofovir disoproxil fumarate (TDF)–associated renal dysfunction may abate when TDF is replaced with abacavir (ABC). The extent to which the third drug atazanavir contributes to renal dysfunction is unclear.

**Methods:**

A retrospective analysis was conducted on adults who had plasma viral load (pVL)<200 copies/mL for≥six months while receiving TDF/lamivudine (3TC) – or TDF/emtricitabine (FTC)–based antiretroviral therapy (ART), then switched to ABC/3TC while retaining the third drug in the ART regimen. CD4, pVL, creatinine, estimated glomerular filtration rate (eGFR), serum phosphorus, urine albumin to creatinine ratio and serum lipids were compared between pre-switch baseline and 3, 6 and 12 months after the switch to ABC.

**Results:**

A total of 286 patients switched from TDF to ABC between 2004 and 2014: 232 (81%) male, median age 48 years (interquartile range (IQR) 42, 56). The third drug was atazanavir (± ritonavir) in 141 (49%) cases. The pVL was<50 copies/mL in 93 to 96% at all time points. Median serum creatinine was 93 µmol/L (IQR 80–111) at baseline and decreased to 88 µmol/L (IQR 78–98) at 12 months after the switch to ABC. Median eGFR increased from 74 (IQR 60–88) mL/min at baseline to 80 mL/min (IQR 69–89) at 12 months. Results were not significantly different between patients on atazanavir versus those on another third drug.

**Conclusions:**

Viral suppression was maintained among patients who switched from TDF/3TC or TDF/FTC to ABC/3TC. Serum creatinine and eGFR improved up to 12 months after switching to ABC/3TC, irrespective of whether or not patients were also receiving atazanavir±ritonavir.

## Introduction

The appropriate use of active antiretroviral therapy (ART) among HIV-1-infected individuals leads to the suppression of viral replication, sustained undetectable plasma viral load (pVL) and CD4 recovery. ART thus halts disease progression to AIDS and premature death and increases life expectancy to near normal levels [1]. Either tenofovir disoproxil fumarate (TDF) or abacavir sulphate (ABC), nucleotide and nucleoside reverse transcriptase inhibitors, respectively, is recommended as part of an initial ART regimen [2]. Recommended and alternative third drug options include integrase inhibitors, non-nucleoside reverse transcriptase inhibitors and the ritonavir-boosted protease inhibitors (PIs), atazanavir and darunavir [3].

ABC is generally safe and well-tolerated, especially since the implementation of HLA-B*5701 allele screening used to predict increased risk for ABC hypersensitivity [3]. TDF use has been associated with renal dysfunction [4–7]. ABC is not known to be nephrotoxic [8,9]; therefore, replacement of TDF with ABC represents a potentially attractive strategy in the setting of renal dysfunction among patients receiving TDF-based ART.

Co-administration of PIs with TDF may be an additional risk factor for renal dysfunction [6,7,10,11]. Among the PIs in current use, atazanavir has been associated with nephrolithiasis [12,13] and chronic kidney disease (CKD) in cohort studies [6,7]. The potential mechanism of the interaction between TDF and atazanavir, as it relates to renal dysfunction, remains unclear [14,15].

TDF has an effect with a possible rebound effect after its discontinuation [16]. In the ASSERT study, treatment-naïve patients receiving ABC/lamivudine (3TC) had greater increases than those receiving TDF/emtricitabine (FTC) in total cholesterol, high-density lipoprotein (HDL) cholesterol, low-density lipoprotein (LDL) cholesterol and triglycerides [17].

We conducted a retrospective study to evaluate changes in serum creatinine, estimated glomerular filtration rate (eGFR) and lipid parameters among HIV-positive adults who switched ART regimens from TDF+3TC or FTC to ABC+3TC, while the remainder of the regimen continued unchanged. We also assessed whether the presence of atazanavir in the regimen affected the aforementioned parameters after the switch.

## Methods

### Study population

We analyzed population-based data from the Drug Treatment Program (DTP) at the British Columbia Centre for Excellence in HIV/AIDS (BC-CfE) from 2004 until 2015. The DTP is a province-wide health care program, which provides free HIV medication to all medically eligible HIV-positive individuals in BC. The BC-CfE collects and de-identifies patient information and uses it for public health monitoring, drug safety, health research and other health system purposes (www.cfenet.ubc.ca/drug-treatment-program). The DTP has received ethical approval from the University of British Columbia ethics review committee at its St. Paul's Hospital site. The program also conforms to the province's Freedom of Information and Protection of Privacy Act.

This study includes male and female DTP participants aged 19 or older, who were receiving TDF/3TC- or TDF/FTC-based ART and had pVL<200 copies/mL for at least six months, prior to switching to an ABC/3TC-based ART regimen, while the third drug in the regimen remained unchanged, regardless of whether the original regimen was a single-tablet regimen. This strategy was used in an attempt to exclude from the analysis patients who changed regimens due to virologic failure, defined as a pVL above 200 copies/mL in two consecutive measurements.

The local standard of care is for all participants to have a documented negative HLA-B*5701 test prior to starting ABC, and to have pVL and CD4 counts measured every three months after starting the new regimen [18]. To ensure adequate follow-up time, the analysis was limited to patients with at least six months of follow-up after the switch to ABC/3TC.

Due to the nature of this retrospective analysis, clinical information, including weight, concomitant medications and history of co-morbidities, was not available for this study.

### Measurements

From the beginning of the current study in 2004 until 31 January 2007, pVL was measured using the Roche COBAS HIV-1 Ampliprep Amplicor Monitor assay ultrasensitive version 1.5 (Roche Diagnostic Systems, Inc., Laval, Quebec, Canada). Starting 1 February 2008, this assay was replaced by the new COBAS Ampliprep Taqman HIV-1 assay (Roche Diagnostic Systems). In view of the documented increased frequency of detectable HIV RNA levels near the lower limit of the Taqman assay (50 copies/mL)[19], for the purposes of study inclusion and this analysis, the lower limit of pVL considered to be clinically significant was set at 200 copies/mL. For consistency, this threshold was used for pVL results obtained throughout the study period.

Renal parameters were assessed within 12 months prior to switching and 3, 6 and 12 months (if available) after switching: serum creatinine, eGFR (calculated using the MDRD formula) [20], serum phosphorus and urine albumin to creatinine ratio (UACR). Fasting total cholesterol, LDL cholesterol, HDL cholesterol and triglycerides were also assessed prior to and 3, 6 and 12 months after switching. Baseline results were defined as those available prior to the switch, using the result closest in time to the switch and within 12 months before switching to ABC. Reference ranges are based on local hospital guidelines [21].

### Statistical analysis

McNemar's test was used on categorical variables and Wilcoxon signed-rank test was used to compare laboratory values before and after the switch. Chi-squared test was used on categorical variables and Wilcoxon rank-sum test was used on continuous variables when comparing atazanavir and non-atazanavir groups. All analyses were performed using SAS software version 9.2 (SAS, Cary, NC) with significance level set at 0.05.

## Results

### Parameters before and after a TDF to ABC switch

A total of 286 individuals were included in the analysis, of whom 232 (81%) were male, median age was 48 years (interquartile range (IQR): 42, 56), 15% had a prior AIDS-defining illness and the median duration of HIV infection was seven years (Table 1). The ethnicity of our cohort was as follows: Asian 5.2% (*n*=15), Black 2.8% (*n*=8), First Nations 11.5% (*n*=33), Hispanic 2.5% (*n*=7), unknown 35.0% (*n*=100) and Caucasian 43.0% (*n*=123). Nearly one-third (*N*=89, 31%) were treatment naïve prior to initiating TDF-containing ART. The TDF-based regimen was started between 2002 and 2014 and switched to an ABC-based regimen between 2004 and 2014. Most patients (95.8%) had pVL<50 copies/mL at their most recent test prior to switching to ABC and this remained the case at 3, 6 and 12 months after the switch (93.2%–94.4%) (Table 2). Median CD4 cell count at baseline was 500 cells/mm^3^ (IQR: 350, 660) and increased after the switch to 550 cells/mm^3^ at 6 and 12 months later (Table 2).

**Table 1:** Baseline characteristics by gender

	Females	Males	Total
***N***	54	232	286
Age, in years, median (IQR)	46 (39, 51)	49 (43, 57)	48 (42, 56)
HIV duration in years, median (IQR)	7 (4, 11)	7 (4, 10)	7 (3.6, 10.5)
CD4 in cells/mm^3^, median (IQR)	480 (360–710)	500 (350–650)	500 (350–660)
Previous AIDS-defining illness, *N* (%)	8 (14.8%)	36 (15.5%)	44 (15.4%)

**Table 2:** Laboratory parameters of total study population (*N*=286) over time before and after switching from tenofovir DF to abacavir

Parameter (reference range) [20]	Baseline on TDF	At 3 months on ABC	At 6 months on ABC	At 12 months on ABC
CD4, cells/mm^3^ (410–1130)	500 (350–660)	500 (350–700)**	550 (398–710)*	550 (410–740)*
pVL<50 copies/mL	95.8%	94.2%	93.2%	94.4%
Creatinine, µmol/L (45–110)	93 (80–111)	90 (79–99)*	89 (77–99)*	88 (78–98)*
eGFR, mL/min (>59)	74 (60–88)	78 (67–90)*	81 (69–91)*	80 (69–89)*
Phosphorus, mmol/L (0.8–1.5)	0.91 (0.74–1.04)	0.94 (0.81–1.06)	0.94 (0.82–1.06)	0.92 (0.77–1.03)
UACR, mg/mmol (<2.0)	2.80 (0.80–10.30)	1.40 (0.90–8.10)*	1.30 (0.70–4.60)*	1.60 (0.80–4.70)*
Total cholesterol, mmol/L (<5.20)	4.35 (3.86–5.17)	5.23 (4.51–5.84)*	5.11 (4.35–5.64)*	4.94 (4.31–5.63)*
LDL, mmol/L (<3.4)	2.52 (2.11–3.17)	2.63 (2.25–3.63)	2.69 (2.26–3.42)**	2.64 (2.14–3.18)
HDL, mmol/L (>0.90)	1.17 (0.97–1.36)	1.22 (1.06–1.51)*	1.21 (1.02–1.56)*	1.22 (1.10–1.52)*
Triglycerides, mmol/L (<1.50)	1.40 (1.00–2.13)	1.86 (1.52–2.97)*	1.62 (1.19–2.65)*	1.86 (1.35–2.73)*

Data shown as median (interquartile range) except plasma viral load (pVL), shown as percentage of the total study population. TDF, tenofovir disoproxil fumarate; ABC, abacavir; eGFR, estimated glomerular filtration rate (MDRD formula); UACR, urine albumin to creatinine ratio.

* *p*<0.001 for change from baseline.

** *p<*0.05 for change from baseline.

Overall, renal parameters improved at each time point after switching from TDF to ABC (Table 2). Serum creatinine decreased, eGFR increased and UACR decreased at every time point compared to baseline. When the participants were grouped by eGFR<60, 60 to 90, or>90 mL/min, there were significant changes over time, with fewer participants in the lowest eGFR category following the switch (Table 3). Median serum phosphorus was in the normal range at baseline and did not change after the switch from TDF to ABC (Table 2).

**Table 3:** Numbers of patients based on eGFR category before and after switch

	Before switch	After switch		
		
eGFR (mL/min)	Baseline, *N* (%)	At 3 months, *N* (%)	At 6 months, *N* (%)	At 12 months, *N* (%)
<60	46 (24.34)	12 (10.43)	20 (11.70	19 (12.42)
60–90	100 (52.91)	75 (65.22)	106 (61.99)	97 (63.40)
>90	43 (22.75)	28 (24.35)	45 (26.32)	37 (24.18)
Total	*N*=189	*N*=115**	*N*=171*	*N*=153**

* *p*<0.001 for change from baseline.

** *p<*0.05 for change from baseline.

Fasting lipid parameters showed small changes after switching from TDF to ABC, as expected (Table 2). Small increases in total cholesterol, HDL cholesterol and triglycerides were observed following the switch from TDF to ABC as compared to baseline. LDL cholesterol was increased at six months but not at the other time points.

### Parameters with or without atazanavir as a third drug

Of the total study population, 49% (*n*=141) were taking atazanavir as the third agent in their ART regimen: 132 ritonavir-boosted (46.1%) and 9 unboosted (3.1%). Of the other 145 patients, 90 (62%) were taking non-nucleoside-based regimens (efavirenz (*n*=43), etravirine (*n*=1), rilpivirine (*n*=2), nevirapine (*n*=44)); 38 (26%) were taking a ritonavir-boosted PI other than atazanavir (amprenavir (*n*=1), darunavir (*n*=4), lopinavir (*n*=31), saquinavir (*n*=2)) and 16 (6%) were taking an integrase inhibitor (raltegravir). There were no significant differences between the atazanavir and the non-atazanavir groups with regard to CD4 cell counts, pVL or renal parameters at either baseline or any time point thereafter, with the exception of serum phosphorus, which was lower (but remained within the normal range) in the non-atazanavir group than in the atazanavir group at 12 months (Table 4). Thus, the presence of atazanavir in the ART regimen did not appear to dampen the beneficial effects on renal function observed after the switch from TDF to ABC.

With regard to lipid changes, triglycerides were higher in the atazanavir group than in the non-atazanavir group at month 12 only (2.21 vs. 1.70 mmol/L, respectively, *p*<0.05). Otherwise, there was no significant difference in lipid parameters between the non- atazanavir and atazanavir groups at any time point (Table 4).

**Table 4:** Laboratory parameters over time before and after switching from tenofovir DF to abacavir, stratified by atazanavir versus non-atazanavir regimens

	Atazanavir (*n*=141)				Non-atazanavir (*n*=145)			
		
Parameter (reference range)	Baseline	At 3 months	At 6 months	At 12 months	Baseline	At 3 months	At 6 months	At 12 months
CD4, cells/mm^3^ (410–1130)	500 (365–650)	510 (360–690)	550 (410–740)	560 (430–730)	490 (340–660)	490 (320–720)	550 (366–700)	530 (370–750)
pVL<50 copies/mL	95.0%	92.2%	92.8%	93.2%	96.6%	96.1%	93.7%	95.6%
Creatinine, µmol/L (45–110)	94 (79–114)	90 (81–101)	89 (75–101)	88 (75–99)	93 (81–108)	87 (77–99)	85 (77–98)	86 (78–98)
eGFR, mL/min (>59)	74 (60–87)	77 (68–88)	81 (70–91)	80 (68–88)	75 (59–89)	78 (66–95)	81 (69–90)	80 (69–91)
Phosphorus, mmol/L (0.8–1.5)	0.90 (0.80–1.06)	0.94 (0.81–1.10)	0.95 (0.83–1.08)	0.95 (0.80–1.05)*	0.92 (0.68–1.01)	0.93 (0.81–1.04)	0.94 (0.81–1.02)	0.88 (0.75–0.96)*
UACR, mg/mmol (<2.0)	2.50 (0.80–8.40)	1.30 (0.70–5.30)	1.30 (0.70–4.60)	1.30 (0.70–4.00)	2.85 (0.80–13.85)	4.0 (1.0–12.3)	1.40 (0.80–4.30)	1.80 (0.90–5.30)
Total cholesterol, mmol/L (<5.20)	4.29 (3.86–5.17)	5.23 (4.62–5.84)	5.18 (4.39–5.73)	5.10 (4.60–5.70)	4.36 (3.93–5.10)	5.25 (4.29–5.72)	5.05 (4.26–5.54)	4.82 (4.10–5.49)
LDL, mmol/L (<3.4)	2.52 (2.13–3.16)	2.65 (2.25–3.21)	2.70 (2.31–3.42)	2.74 (2.23–3.26)	2.55 (2.06–3.26)	2.60 (2.21–3.80)	2.69 (2.09–3.39)	2.63 (2.02–3.04)
HDL, mmol/L (>0.90)	1.19 (1.04–1.34)	1.25 (1.08–1.52)	1.20 (1.05–1.52)	1.21 (1.10–1.48)	1.08 (0.96–1.40)	1.17 (1.02–1.41)	1.21 (1.02–1.59)	1.24 (1.08–1.54)
Triglycerides, mmol/L (<1.50)	1.47 (1.04–2.17)	2.26 (1.58–3.05)	1.85 (1.26–2.66)	2.21 (1.47–3.08)*	1.29 (0.90–2.00)	1.71 (1.46–2.80)	1.40 (1.14–2.57)	1.70 (1.14–2.32)*

Data shown as median (interquartile range) except plasma viral load (pVL), shown as percentage of study population in each group.

* *p<*0.05 for comparison between groups.

## Discussion

We conducted a retrospective analysis of 286 HIV-infected patients who were switched from a TDF- to an ABC-based ART, while continuing the rest of antiretroviral drugs in the regimen. Our results show a modest, but significant, improvement in serum creatinine, eGFR and UACR results up to 12 months after the switch, without significant changes in the proportion of patients with undetectable plasma HIV RNA after the switch to ABC.

The median baseline eGFR in the study group was 74 mL/min, indicating some degree of kidney impairment according to standard definitions [22]. Whether or not this is due to the TDF-containing ART regimen is unclear; however, previous literature outlining the negative effects of TDF on renal function should be considered [22–24]. Retrospective and observational studies suggest that TDF may induce subclinical nephrotoxicity, which mostly involves the proximal tubule [5,24].

It has been reported that HIV-positive patients exposed to TDF-based regimens have a higher incidence of chronic renal dysfunction that in many cases is reversible once TDF is discontinued [22], but not in all cases [25]. In a Japanese observational study, antiretroviral-naïve patients that started TDF-containing therapy experienced eGFR decline twice as often as those treated with an ABC-containing regimen [8]. Interestingly, in our study, we demonstrate that even in those patients experiencing mild abnormalities in renal function, there is an improvement of the renal parameters once TDF is discontinued, regardless of the other components of the ART regimen.

Other antiretrovirals, particularly the PIs indinavir and atazanavir, have also been associated with renal function abnormalities [4]. In a study by Calza *et al*. [26] patients receiving TDF/FTC and atazanavir/ritonavir for 12 months had a larger decline in eGFR and more proximal tubulopathy than those receiving TDF/FTC plus efavirenz or lopinavir/ritonavir. Furthermore, in the D:A:D study, use of TDF, ritonavir-boosted atazanavir and ritonavir-boosted lopinavir were independent predictors of chronic renal impairment in the absence of pre-existing renal impairment [7]. It is important to note that there are a number of other independent predictors of CKD, such as diabetes, hypertension, concomitant nephrotoxic medications and smoking. Unfortunately, due to the limitations of our database we were unable to capture these factors in our analysis.

In this retrospective analysis, patients receiving atazanavir had no statistically different changes in their serum creatinine and eGFR after the discontinuation of TDF as compared to those not receiving atazanavir. In a retrospective analysis of 230 patients, half of which received TDF and the other half ABC-based therapy, a significant renal impairment in the patients receiving TDF was observed, but there was no correlation to PI use [27]. Furthermore, use of TDF was the only independent predictor of progression to CKD stages 2 and 3 in HIV-infected patients after controlling for risk factors including diabetes mellitus, hypertension, chronic hepatitis C infection, the use of non-nucleosides, concomitant administration of sulfamethoxazole/trimethoprim, or non-steroidal anti-inflammatory drugs.

With respect to the lipid parameters, our results confirm the earlier findings of an increase in lipids after TDF discontinuation [16], although the clinical significance is likely to be small. However, dyslipidemia is a common problem among the HIV population due to a multitude of factors and should be addressed as an important part of their clinical management [28].

We recognize this study has several limitations. Firstly, this is not a randomized controlled trial and given its retrospective nature does not allow for comparison with a control group and may be affected by selection bias. Next, we could only assume the reason why the patients were switched from TDF to ABC based on the standard of care in our clinic. Because of the retrospective nature of this analysis, we were unable to examine novel indicators of proximal renal cell dysfunction, like urinary excretion of phosphate, glucose, uric acid or the fractional excretion of phosphate [29]. Finally, this study has a limited sample size and a male predominance, thus limiting the number of feasible sub-analyses.

## Conclusions

In conclusion, a significant improvement of the serum creatinine, eGFR and UACR was observed in 286 HIV-positive patients who were switched to ABC after receiving a TDF-based ART regimen for at least six months. Of note, after the switch, the plasma HIV RNA remained suppressed and the CD4 cell count increased in the majority of patients. Similar trends were observed regardless of whether or not the third drug in the regimen was atazanavir. These findings highlight that it is safe and effective to switch from TDF to ABC if there are concerns regarding serum creatinine and eGFR, as long as the HLA-B*5701 test is negative and the patient's HIV RNA viral load is suppressed. Finally, the continued use of atazanavir did not appear to have a significant effect on the recovery of the renal function parameters.
